# Quantifying Plant Colour and Colour Difference as Perceived by Humans Using Digital Images

**DOI:** 10.1371/journal.pone.0072296

**Published:** 2013-08-20

**Authors:** Dave Kendal, Cindy E. Hauser, Georgia E. Garrard, Sacha Jellinek, Katherine M. Giljohann, Joslin L. Moore

**Affiliations:** 1 Australian Research Centre for Urban Ecology, Royal Botanic Gardens, Melbourne, Victoria, Australia; 2 School of Botany, University of Melbourne, Parkville, Victoria, Australia; 3 School of Global, Urban and Social Studies, RMIT University, Melbourne, Victoria, Australia; CSIC-Univ Miguel Hernandez, Spain

## Abstract

Human perception of plant leaf and flower colour can influence species management. Colour and colour contrast may influence the detectability of invasive or rare species during surveys. Quantitative, repeatable measures of plant colour are required for comparison across studies and generalisation across species. We present a standard method for measuring plant leaf and flower colour traits using images taken with digital cameras. We demonstrate the method by quantifying the colour of and colour difference between the flowers of eleven grassland species near Falls Creek, Australia, as part of an invasive species detection experiment. The reliability of the method was tested by measuring the leaf colour of five residential garden shrub species in Ballarat, Australia using five different types of digital camera. Flowers and leaves had overlapping but distinct colour distributions. Calculated colour differences corresponded well with qualitative comparisons. Estimates of proportional cover of yellow flowers identified using colour measurements correlated well with estimates obtained by measuring and counting individual flowers. Digital SLR and mirrorless cameras were superior to phone cameras and point-and-shoot cameras for producing reliable measurements, particularly under variable lighting conditions. The analysis of digital images taken with digital cameras is a practicable method for quantifying plant flower and leaf colour in the field or lab. Quantitative, repeatable measurements allow for comparisons between species and generalisations across species and studies. This allows plant colour to be related to human perception and preferences and, ultimately, species management.

## Introduction

Quantifying plant colour as perceived by insects and animals has made a significant contribution to our understanding of pollination ecology [1], foraging theory [2] and the evolution of plants [3]. How humans perceive colour has received much less attention but recent work suggests it too has important ecological implications. In conservation ecology, plant colour influences the detectability of invasive species, and rare and threatened species [4]. This is important because detection rate is a key parameter when designing surveys [5,6], managing cryptic species [7,8], designing monitoring programs [9,10] and managing invasive species [11,12].

Plant colour as perceived by humans is typically measured and recorded using subjective categorisation rather than quantitative measures in studies of plant detectability [4], trait databases [13,14] and in studies of human plant selection [15]. This limits the usefulness of plant colour traits in biodiversity and conservation management applications such as species detection modelling. Subjective categorisation of colour constrains the interpretation of findings to the system at hand, as categories are likely to vary between systems and observers. In contrast, a transparent, robust and repeatable method for measuring plant colour allows generalisation of findings across systems, and can improve models of the influence of colour on detectability. Quantification will also allow colour difference to be calculated. Colour contrasts between species have been shown to be important in foraging studies [16], and it seems likely that the difference in colour between a target species and the surrounding vegetation will be a useful predictor of detectability. Such colour contrasts are likely to vary across a landscape and are difficult to measure qualitatively.

Methods have been developed to quantitatively measure plant and animal colour using digital images in the study of animal coloration [17], determining vegetation cover [18–21] and monitoring coral reef health [22]. Digital images have also been widely used to measure plant colour in agricultural studies, where fertility is assessed using leaf colour [23], and fruit and vegetables are sorted for ripeness by measuring colour [24]. There are also a number of studies that quantify plant and animal colour with reference to a particular vision system, such as from the perspective of insect vision systems in pollination studies [25] and animal (particularly bird) vision systems in studies of foraging and display [26,27]. The main advantage of using a particular vision system is that the colour contrast as perceived by a particular animal group can be determined. Like all old-world primates, humans have trichromatic colour vision with photo receptors sensitive to peak wavelengths of approximately 560 nm, 535 nm and 430 nm [28]. Colour discrimination is most acute where spectral response of photoreceptors overlap [29]. Humans are therefore particularly sensitive to differences between red and green colours where there is a large spectral overlap in photo-receptors.

Digital images are typically recorded as a raster of 3-colour (red/green/blue or RGB) pixels. All possible colours can be represented in a cubic space. However, differences in colour in RGB space are not closely related to human perception of colour difference, and RGB values are typically not standardised (RGB values depend on the instrument used to capture the image [30]:). RGB data are often standardised and transformed into different colour spaces more suited to a particular application. The International Commission on Illumination (CIE) has defined a number of standard colour spaces that are widely used. The CIE 1976 (L*a*b*) colour space is a standardised (device-independent) non-linear transformation of the RGB colour space [31] modelled on human perception of colour [30]. It has linear measures of lightness (L*) and two colour dimensions (a* and b*) that are suitable for use in mathematical models. The a* dimension represents a spectrum from green (negative) to magenta (positive) and the b* dimension represents a spectrum from blue (negative) to yellow (positive).

Colour can be measured using a variety of instruments such as spectrometers [32] and digital cameras [21]. As the image quality of digital cameras has improved markedly in recent years, their affordability and ready availability make them a desirable and suitable tool for field data collection. Measurements can be made in the field or in the lab from material collected in the field. However, it is not clear if all cameras produce images of suitable quality for plant colour measurements.

This paper presents a standard method for measuring plant colour using digital images. We demonstrate this by 1) assessing the viability of measuring plant leaf and flower colour in the field and 2) measuring the relative accuracy of colour measurements using a range of digital cameras. Applications of these plant colour measurements are then explored by calculating 3) differences in flower colour between species and 4) yellow-orange flower cover within vegetation quadrats.

## Materials and Methods

### Capturing images of plant colour

We collected and analysed data from two separate studies. In the first experiment, flower colour data were collected from hand-crafted models of two invasive species, the yellow flowered *Hieracium* *praealtum* Vill. ex Gochnat and orange flowered *H* *. aurantiacum* L., specially constructed for the purposes of the experiment. Flower colour data were also collected in the field for nine flowering species with similar flower colours (yellow to orange) and sizes/shapes (1 cm to 3 cm in diameter that were generally round) as part of an experiment exploring the detectability of the two invasive species in sub-alpine grasslands near Falls Creek, Australia (36°51'48″S 147°16'54″E). Data on the proportional cover of yellow-orange flowers in field plots was also collected. In the second experiment designed to test the reliability of the method, leaf colour data were collected from five different shrub species from a residential garden in Ballarat, Australia (37°33'44″S 143°50'46″E) using five different digital cameras.

Flower colour data was collected by photographing all yellow-orange flowering species (File S1) present in sixteen 20 m x 20 m field plots using a good quality digital SLR (Nikon D300) and a slightly wide angle lens (36 mm equivalent). Following the protocols outlined by Cornelissen et al. [33] for collecting trait data, at least five and preferably ten flowers of at least five haphazardly selected individuals of each species were photographed (Table 1). In each plot, yellow-orange cover was estimated in 1 m^2^ quadrats (n=25), and a photograph (total n=400) was taken of each quadrat (File S2). Yellow-orange cover photographs were taken from as close as practicable to directly overhead using the available digital cameras (Nikon D300, Sony NEX-5n, Canon Powershot A720) with a slightly wide angle lens focal length (36 mm equivalent). This focal length allowed the 1 m^2^ quadrats to be photographed with the cameras held by hand rather than using tripods and ladders. To compare digital imaging methods with more traditional methods of cover estimation, the number of flowers of each yellow-orange flowering species was counted. In each quadrat, the major and minor orthogonal diameters were measured for one randomly selected (by tossing a coin) flower of each species. Each sampled flower was treated as an ellipse to calculate its visible area (Area = π × major radius × minor radius), then flower area was averaged across samples within a species. Total yellow-orange cover area was simply calculated by multiplying mean flower area by flower count for each species, then summing over all species. Leaf data were collected by photographing ten leaves of each shrub species (File S3) using five different cameras: a digital SLR (Nikon D300 using a 36 mm equivalent lens), a mirrorless camera (Sony NEX-5n zoomed to a 36 mm equivalent lens), a compact digital camera (Ricoh CX4, zoomed to 28 mm equivalent lens), a phone camera (HTC Incredible S) and a tablet camera (Samsung Galaxy Tab 7.7).

**Table 1:** Summary statistics for species measured in this study.

			**Colour means**			**Kolmogorov-Smirnov statistic**		
**Species**	**Part**	**N**	L*	a*	b*	L*	a*	b*
*Bulbine* *bulbosa* (R.Br.) Haw.	flower	13	57.1 ±1.5	4.4 ±1.5	62.2 ±1.4	0.11	0.10	0.10
*Craspedia* *aurantia* - *C* *. jamesii* complex J. Everett & Joy Thomps.	flower	14	59.4 ±2.6	25.2 ±3.2	65.8 ±2.2	0.11	0.14	0.14
*Craspedia* *coolaminica* J. Everett & Joy Thomps.	flower	17	62.4 ±3.0	23.8 ±3.0	67.9 ±2.5	0.12	0.12	0.15
*Hieracium* *aurantiacum* L.	flower	14	61.5 ±2.6	39.0 ±2.5	62.6 ±1.9	0.11	0.15	0.12
*Hieracium* *praealtum* Vill. ex Gochnat	flower	14	77.9 ±2.2	8.0 ±1.7	78.7 ±1.8	0.11	0.10	0.19**
*Helichrysum* *rutidolepis* DC.	flower	12	53.8 ±5.0	10.0 ±2.9	56.6 ±11.1	0.11	0.12	0.16
*Hypochaeris* *radicata* L.	flower	10	64.1 ±2.7	11.1 ±3.4	68.1 ±1.2	0.10	0.16	0.11
*Kunzea* *muelleri* Benth.	flower	10	60.7 ±4.3	-7.2 ±1.3	42.2 ±6.1	0.14	0.11	0.16
*Microseris* *lanceolata* (Walp.) Sch. Bip.	flower	5	69.7 ±2.9	2.5 ±2.1	70.0 ±3.4	0.10	0.08	0.12
*Ranunculus* *victoriensis* B. G. Briggs	flower	5	62.6 ±3.3	-0.7 ±1.0	66.5 ±2.8	0.13	0.10	0.19**
*Senecio* *pinnatifolius* var. *alpinus* (Ali) I. Thomps.	flower	11	65.4 ±2.9	6.0 ±2.0	69.2 ±2.3	0.13	0.13	0.10
*Arctotis* * × hybrida* hort.	leaf	20	43.1 ±6.7	-14.8 ±7.5	18.6 ±4.9	0.11	0.17	0.12
*Buxus* *sempervirens* L.	leaf	20	23.3 ±4.6	-8.2 ±2.2	5.8 ±3.1	0.11	0.10	0.12
*Callistemon* *rugulosus* (D. F. K.Schltdl. ex Link) DC.	leaf	20	30.1 ±4.3	-12.4 ±4.3	12.2 ±4.8	0.12	0.10	0.12
*Eucalyptus* *kitsoniana* Maiden	leaf	20	33.6 ±4.1	-10.8 ±3.9	15.8 ±3.9	0.15	0.19	0.16
*Pittosporum* *tenuifolium* Gaertn.	leaf	20	38.1 ±3.4	-17.4 ±4.1	23.7 ±3.1	0.13	0.17	0.14

The number of leaves or flowers measured of each species, and the Kolmogorov-Smirnov statistic comparing L*, a* and b* distributions with a Gaussian distribution modelled from the mean and standard deviation are also shown. ** the null hypothesis that the actual and modelled distributions are the same is not supported at P<0.05.

All photographs used camera settings that provided the highest image quality. The highest pixel count was used to provide the maximum amount of colour information (most pixels per object area). The lowest sensitivity (ISO) settings were used to maximise signal to noise ratio. The images were underexposed by at least 0.3 stops to minimise colour clipping, which occurs when RGB channels reach their maximum value and results in the loss of colour information [34]. RAW file capture was used on cameras where it was available. RAW files contain the data captured by the image sensor with relatively little processing. In contrast, images captured as JPEG files are processed in camera and compressed, losing information in the process.

The colour of an object is greatly influenced by the colour and brightness of the light source used to illuminate it. To minimise variation in light colour and brightness, photographs were taken in the middle of the day when the sky was overcast to minimise shadows and fluctuations in colour temperature that can occur in early morning and evening light. To further compensate for variations in light source colour and brightness and allow captured colour and brightness information to be standardised and transformed into device-independent CIE 1976 (L*a*b*) space in post-processing, a ColorChecker colour rendition chart [35] was included in all images. This allows post-production colour and exposure balancing of the images.

### Image colour standardisation

The RGB colour space used by digital cameras is device-dependent. Measured RGB colour values are related to the colours of the objects being recorded, the sensitivity of the recording image sensor, and prevailing lighting conditions (colour and brightness). A method of standardisation is required before images can be analysed [27]. This is typically achieved by including a reference object (such as the ColorChecker chart) in the image, then adjusting four image parameters during image post-production so captured values match predefined values for the reference object. Colour temperature adjusts the relative sensitivity of colours on the blue to yellow axis; tint adjusts the relative sensitivity of colours on the magenta to green axis; exposure adjusts the overall brightness level; and black point adjusts the absolute lowest light level recorded. For each captured image, exposure and black point were adjusted so that the RGB values of the ColorChecker neutral (black, greys and white) squares approximated expected RGB values [36]. Sensor non-linearities make it difficult to colour balance across the brightness spectrum using standard software [27], so colour balance was fine-tuned for brightness levels similar to the plant part being measured. Colour temperature and tint were adjusted so that the RGB values of the grey square most similar in brightness to the object being measured closely matched expected RGB values. These adjustments were made using the open source software RawTherapee v 4.08 [37].

### Measuring colour traits

Flowers and leaves were isolated from the image background using the Color Threshold function in the open source image analysis software ImageJ v 1.45 [38]. Flower and leaf images were transformed into CIE 1976 (L*a*b*) space using the *convertColor* function in the *grDevices* package in R v 2.15 [39] (see file S4 for R code). This results in values between 0 and 100 for the L* dimension, and -100 and 100 for the a* and b* dimensions. The suitability of three different distribution functions (Gaussian, Skew-Normal and Beta) for modelling CIE 1976 (L*a*b*) colour were compared to determine whether two parameters (mean and standard deviation), three parameters (mean, standard deviation and skewness) or four parameters (mean, standard deviation, skewness and kurtosis) were required to adequately model L*, a* and b* colours. Skewness and kurtosis were calculated using the *skewness* and *kurtosis* functions in the *moments* package in R. A Kolmogorov-Smirnov test (using the *ks.test* function in the *stats* package in R) was used to determine whether the modelled distribution functions were significantly different from the actual colour distributions. Intra-specific variation in colour measurements was explored using a non-metric multidimensional scaling (nMDS) of Euclidean distance in three dimensional CIE 1976 (L*a*b*) space. This was performed separately for flowers and leaves using the *isoMDS* function in the *MASS* package in R v 2.15. Non-metric multidimensional scaling is a technique used to visually represent the similarity of non-linear multidimensional data in two dimensional space [40]. An nMDS was also used to compare the similarity of colour measurements from different cameras.

### Measuring colour difference

Colour difference between yellow-orange flowering species was calculated using the Earth Mover’s Distance (EMD), a measure of the difference between two distributions that is commonly used in image processing [41]. Conceptually, the Earth Mover’s Distance is a measure of the minimum amount of effort required to transform one distribution into another: “Intuitively, given two distributions, one can be seen as a mass of earth properly spread in space, the other as a collection of holes in that same space. Then, the EMD measures the least amount of work needed to fill the holes with earth” [41: p104]. The mean and standard deviation of the Earth Mover’s Distance was calculated between the colour of the two target *Hieracium* species and each of the nine yellow-orange flowering species using the *emd* function in the *emdDist* package in R. Earth Mover’s Distance was calculated on 3-dimensional distributions in CIE 1976 (L*a*b*) space.

### Measuring yellow-orange cover

To provide square images with an even distribution of pixels across the quadrat, quadrat photos were deskewed, rotated and cropped in GIMP, an open source image editing software program [42]. Yellow-orange flowers were isolated from the background using ImageJ’s *Color Threshold* function described above. Proportional yellow-orange cover was then calculated as the number of yellow-orange flower pixels divided by the total number of quadrat pixels.

### Ethics statements

The sub-alpine grassland field work in this study was carried out on public land. Permission to access this land was granted by the Falls Creek Resort Management, the statutory authority managing the land. The residential garden field work in this study was carried out on private land, and permission to access the land was granted by the property owner.

## Results

### Plant colour measurements

**Figure 1:**
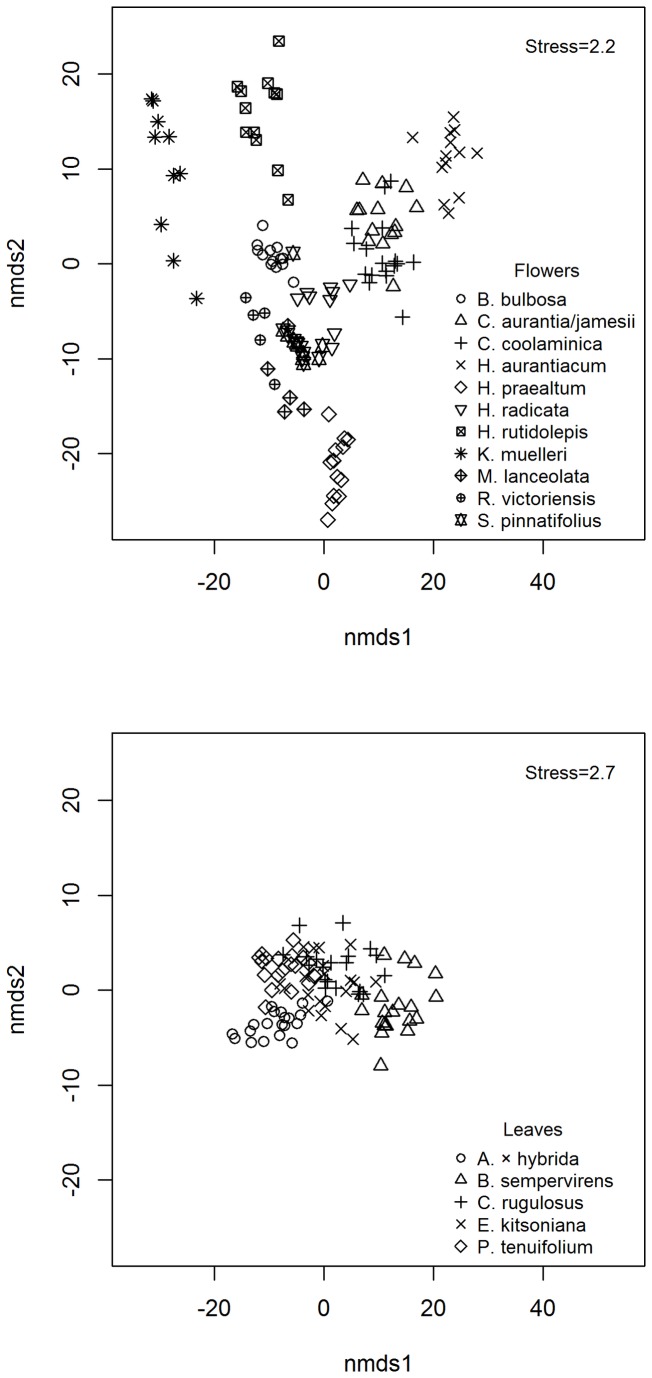
Leaf and flower colour difference. Pairwise Euclidean distance in CIE 1976 (L*a*b*) space was calculated and an nMDS generated for a) yellow-orange flowers and b) leaves (the leaf nMDS used data from the two highest quality cameras only: the Nikon D300 and Sony NEX-5n).

Colour measurements show more inter-species variation in flower colour than leaf colour (Table 1, Figure 1). As expected for yellow-orange flowers, most mean b* values (yellowness) were high, although two species ( *Helichrysum* *rutidolepis* DC. and *Kunzea* *muelleri* Benth.) were clearly less yellow than the other species, and had correspondingly lower mean b* values. There was less variation in leaf colour; all had lower a* (more green) and b* (less yellow) values than the yellow flowers. *Arctotis* *× hybrida* hort. had higher L* values (brightness), while *Buxus* *sempervirens* L. had lower L* (brightness) and b* (yellow) values. The flower colours of most species were easily separated in CIE 1976 (L*a*b*) space except for the two *Craspedia* species (Figure 1). While there was some separation of species leaf colour in CIE 1976 (L*a*b*) space, this was much less pronounced than for flowers.

Comparisons between the actual L*, a* and b* colour distributions and two parameter Gaussian models based on the mean and standard deviation showed that there were mostly no significant differences between actual and modelled distributions (Kolmogorov-Smirnov statistic P<0.001), and that therefore the Gaussian function was an adequate model of colour distribution (Table 1, Files S5 & S6). The only significant differences were in the flowers of *H* *. praealtum* and *Microseris* *lanceolata* (Walp.) Sch. Bip, which had relatively pure and narrow b* colour distributions (File S5). The three and four parameter models (results not shown) did not fit the observed data better than two parameter models.

### Colour difference

**Figure 2:**
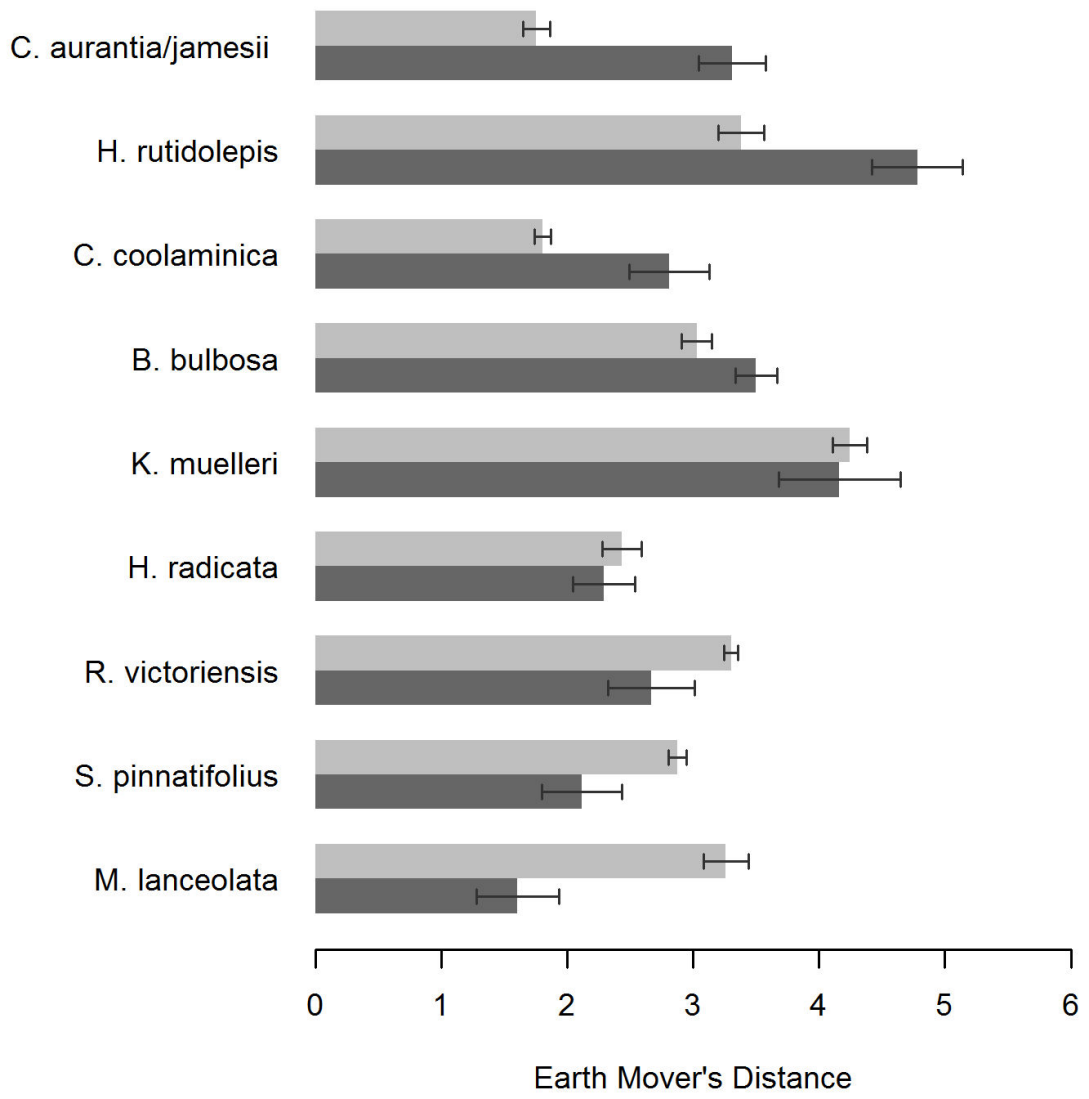
Flower colour difference between species in the field and two invasive species. Pairwise mean flower colour difference (measured as the Earth Mover’s Distance) was calculated between individual yellow-orange flowers of different species and mean colour of individuals of *H*. *aurantiacum* (light bars) and *H*. *praealtum* (dark bars). 95% confidence intervals are shown.

Colour difference as measured by Earth Mover’s Distance showed that some species were more similar to *H* *. praealtum* (e.g. *M* *. lanceolata* *,* *Senecio* *pinnatifolius* var. *alpinus* (Ali) I. Thomps.), while others were more similar to *H* *. aurantiacum* (e.g. both *Craspedia* spp., *H* *. rutidolepis* ) (Figure 2). Multiple comparisons using Tukey’s HSD test (as the data were normally distributed) showed significant (P<0.05) differences between the two target species and all species except *Hypochaeris* *radicata* L., *Ranunculus* *victoriensis* B. G. Briggs and *K* *. muelleri* . These results were largely consistent with qualitative assessments of colour, e.g. the yellow flower of *H* *. praealtum* was more similar to the lemon flowered *M* *. lanceolata* , while both *Craspedia* species have a more orange tint to the flowers that was more similar to the flowers of *H* *. aurantiacum* (File S1).

### Yellow-orange cover

There was a high correlation (R^2^=0.79) between yellow-orange cover measured from digital images and yellow-orange cover estimated from flower counts and diameters. However, cover measured from digital images was systematically lower (regression slope = 0.56) than cover measured by counts and diameters.

### Camera variation

**Figure 3:**
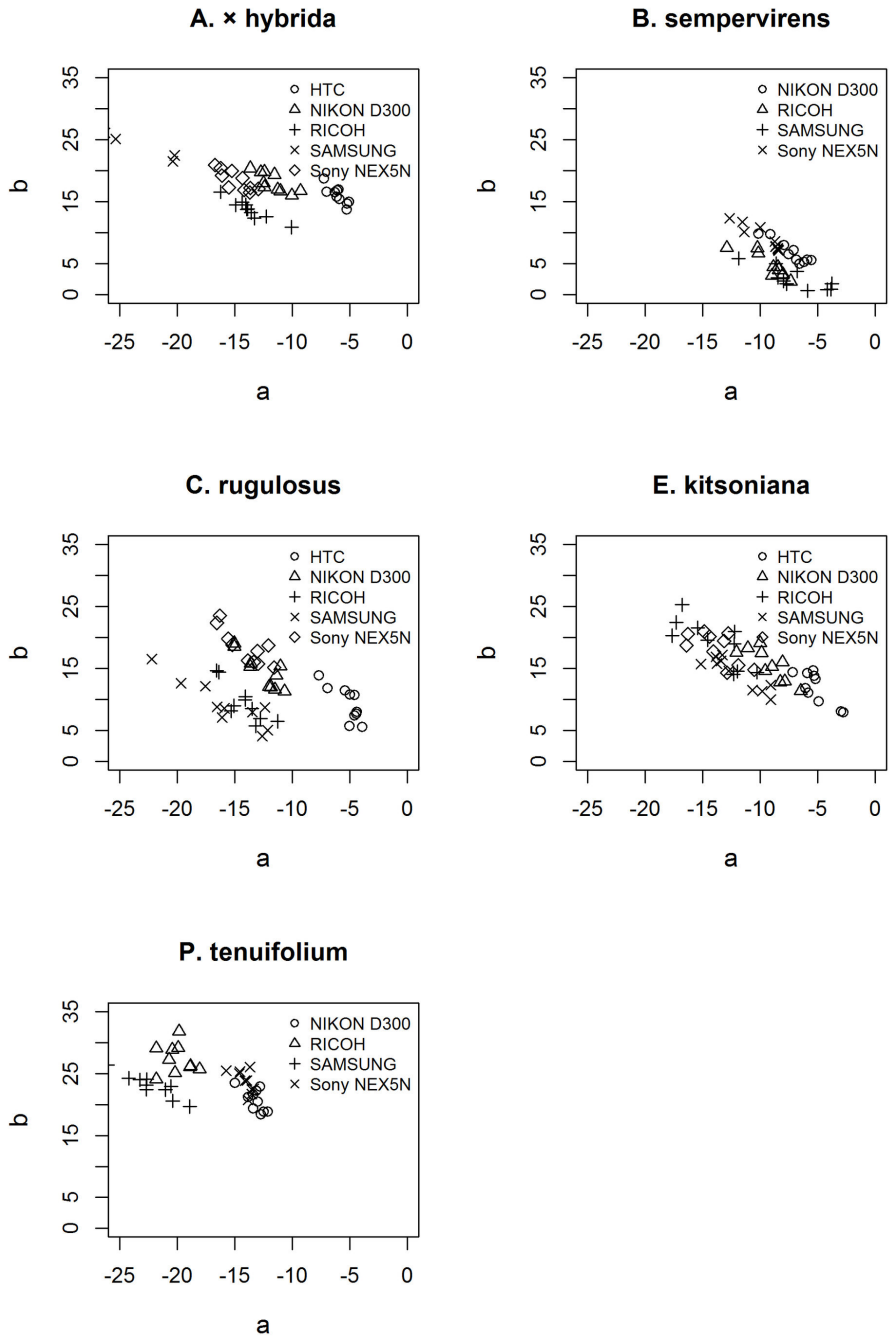
Variation in leaf colour measured by different cameras. Leaf colour is shown in a*-b* space for five species calculated from images taken with five different digital cameras.

There was clearly some systematic variation in colour measurements from different cameras, despite careful standardisation of images (Figure 3). The measurements from the two cameras with larger sensors (the Nikon D300 and Sony NEX-5n) were similar, although the Nikon D300 consistently had slightly higher a* values, while measurements from the Sony NEX-5n had slightly higher b* values. The measurements from the other cameras (Ricoh CX4, HTC Incredible S and Samsung Galaxy Tab 7.7) were more inconsistent.

## Discussion

The digital camera based method presented in this study is well suited for measuring plant colour traits and colour difference in the field and in the lab, although there is some variation based on the quality of the camera used. This method can be used for a variety of applications, such as determining the quantitative difference between colours of different species, or measuring cover of a particular vegetative colour.

### Plant colour measurements

Flower and leaf colours were able to be measured repeatedly, and with a degree of accuracy that allowed different species to be distinguished in CIE 1976 (L*a*b*) space (more so for flowers than for leaves). Colour differs from other traits commonly measured in ecology as it is a multidimensional value, and each measurement includes many individual points (pixels) rather than a single value. As a consequence, analysis of colour is well suited to distribution based analysis (e.g. Bayesian, Earth Mover’s Distance). However, for some applications, such as recording colour as a standardised trait, summary statistics are more useful. The two parameters (mean and standard deviation) of L*, a* and b* values modelled actual colour distributions well in this study. Where leaf and flower parts were a uniform colour, the modelled values were excellent approximations of actual colour distributions. However, models were less accurate where there was colour variation within individual leaves or flowers. Where flowers and leaves have very different colours, such as variegated leaves, or very differently coloured flower parts, it may be appropriate to measure and record the colour values of each component separately. While single parameter (mean) measurements are commonly used for recording traits in databases [13,14], recent studies have shown that intra-specific trait variation can be as large as inter-specific variation [e.g. 43], and it may be appropriate to record traits as two-parameter measurements to improve the quality of information recorded.

A quantitative and objective measure of plant colour has the potential to improve plant management through improved estimation of plant dynamics and improved modelling of human-plant interactions. Plant colour and colour difference are key components of detectability, important for the detection of rare and threatened species during flora surveys [4] and the detection of invasive species by land managers. Understanding plant detectability during surveys allows managers to adjust survey estimates according to local conditions, such as target contrast with background vegetation, prevailing light levels, weather conditions and similarity to previously studied species. This leads to better quality information being used in environmental management and decision-making. Categorical data can be used in a particular survey scenario, such as comparing the relative detectability of flowering *H* *. praealtum* and *H* *. aurantiacum* in the presence of other yellow-orange flowering species. However, the use of quantitative measures of plant colour allows extrapolation to other survey scenarios. Recording plant colour in trait databases could allow colour-based detectability models to be developed as a desktop exercise, leading to improved management planning and resource allocation. This will be useful to conservation planning and management as detectability experiments are resource-intensive and are rarely conducted for species of interest to environmental managers. Colour is one of a number of factors that influence human perception of a natural scene. Detectability is also likely to be affected by shape, contrast, background complexity and texture, among other things. Quantifying colour will allow us to develop more accurate models that can tell us the relative importance of these different factors.

More broadly, this study is relevant to understanding the role that humans play in shaping ecological systems [44]. Colour is important in human perception of plants, and may play a role in human decision making about plants, such as which species to cultivate [15], or which species to transport. By quantifying those colours most preferred by humans, human preference can be included as a predictive factor in models that describe, for example, the composition of different plant communities, or the distribution of particular species. In urban systems, colour may be an important factor differentiating human perception of native and cultivated plant communities, and consequently a factor influencing the acceptability of ecological restorations [45]. It has also been argued that colour is an important ecosystem property in its own right that needs to be considered in conservation [46]. Quantitative measurement of colour will allow this property to be measured and managed, for example by setting benchmarks. Plant colour is also a trait of interest to other disciplines such as environmental psychology, where plant colour has been shown to elicit affective and cognitive responses in individuals [47]. As such, quantitative methods for measuring plant colour are likely to be of broad interest to researchers across a variety of disciplines.

Quantitative measurement of plant colour may also have uses that do not reference human perception. For example, leaf colour may vary along environmental gradients in much the same way as other traits such as leaf width and specific leaf area [48]. The CIE 1976 (L*a*b*) colour space has been shown to be a suitable system for measuring plant nutrient status [23]. However, the CIE 1976 (L*a*b*) colour space may not be suited for measuring colour important to other animal species. While plant colour is an important driver of pollinator interactions, pollinators often use vision systems that are quite different from humans. For example Hymenopteran (e.g. bees) vision is based on blue, green and ultra-violet photoreceptors [25].

There are several advantages of the CIE 1976 (L*a*b*) colour space for measuring plant colour traits using digital cameras. Device independence means that different cameras can be used for measurements and, after standardisation, produce similar results. Linear measures of colour can be used in mathematical models for a variety of purposes (e.g. detectability). Perceptual uniformity means that colour difference can be measured in ways that are relevant to human perception. However, human colour vision is not completely homogenous. Cultural and language-based differences in human colour perception [49,50] may influence colour perception in ways that are not modelled by the CIE 1976 (L*a*b*) colour space. For example, the Greek language has words distinguishing dark and light blue, and experiments show that native Greek speakers can distinguish these colours faster and more accurately than English speakers [51]. Similarly, the colour vision of people who are colour-blind, which results from a person missing types of photo-receptors or having photo-receptors that respond to a different frequency than colour-normal people [52], is not as well modelled as people with colour-normal vision. While the techniques presented here allow quantification and standardisation of colour and colour difference, further research is required to understand the between-subject variation in the application of the models developed using these techniques (e.g. variation across different observers undertaking weed eradication).

### Colour difference

Earth Mover’s Distance proved to be a feasible quantitative measure of colour difference that was consistent with subjective categorisation (File S1). As a distribution based calculation, it retains more colour information than Euclidean distance calculated from mean L*, a* and b* values in Cartesian space. This quantitative measure of colour difference provides a method for measuring the size of the difference between two colours. Intuitively, greater contrast between background vegetation and a target plant will increase the detection probability of the individual. However, the lack of a simple way to quantify the difference between colours has made it difficult to study how colour contrast affects detectability. This measure offers the opportunity to better understand how colour contrast affects detection and build a predictive model of plant detectability [4].

### Yellow-orange cover

Yellow-orange cover calculated from digital images also showed a good correlation with measurements manually calculated from the number and diameter of flowers. The systematic difference between the two techniques can be explained by the way the measurements are made. Firstly, the manual method assumes that the face of the flowers is always perpendicular to the observer, while in reality this is often not the case. The flower area measured by the camera includes only the area visible to the camera, and will be systematically less than the manual calculations. Secondly, the manual method assumed flowers were elliptical with size measured in two dimensions while the camera method doesn’t make any assumptions about shape. Lastly, the classification of flowers as yellow-orange was more subjective in the manual method – and some species, or individual senescing flowers that were included in the manual count may not have been detected as yellow-orange by the digital image threshold approach.

### Reliability across cameras

The quality of the camera taking the images affected measurement results. Similar measurements were attained from the two cameras (the Nikon and the Sony) with larger sensors (approx 370 mm^2^), although there was a small systematic difference between them. The measurements from the smaller sensor camera (Ricoh CX4, approx 30 mm^2^), phone (HTC Incredible S, approx. 15 mm^2^) and Samsung Galaxy Tab 7.7 (sensor size unknown) were less consistent than the larger sensor cameras. There are a number of factors that may explain these discrepancies. The Nikon and Sony have larger individual photo-sites that are able to collect more photons per pixel, and consequently have lower signal to noise ratios, and are able to record colours more accurately [53]. They also can record images in RAW mode where sensor data can be captured with relatively little in-camera processing and in a format that retains all pixel information [27]. In contrast, the other cameras tested record images after extensive in-camera processing (such as sharpening and noise reduction) and as JPEGs, which use a highly compressed file format that does not retain all image data. Lastly, non-linearities in sensor response may make calibration extremely difficult using standard software tools [27]. Camera quality does make a difference, although most currently available digital SLRs and mirrorless cameras should be suitable for measuring plant colour. Current phone cameras and small sensor compact point-and-shoot cameras are less reliable and should be avoided if possible. However, rapid improvements in phone and tablet camera quality make these likely to be more suitable for colour measurement in the near future. This is a desirable outcome as tablets are becoming more widely used in ecological fieldwork.

While the pixel count of the better quality cameras is higher (12-16 MP) compared to the lower quality cameras (3-8 MP), this is not necessarily relevant for measuring colour. For large leaves and flower parts, pixel count will be irrelevant as the object may cover thousands or millions of pixels. However for very narrow leaves or flower parts, pixel count may be important. To minimise edge effects the width of the smallest object to be measured should be at least several pixels across [27]. For example, an image of a leaf 1 mm wide on a background that is 500 mm wide would be more than 6 pixels wide on the Sony Nex-5n, but only 3 pixels wide on the Samsung.

A big advantage of higher quality digital cameras is their performance in poor lighting conditions. Better quality cameras are generally able to record scenes with a higher dynamic range (the difference between the brightest and darkest objects able to be recorded), record scenes in low light with less sensor noise, have more accurate in-camera colour balancing and more flexible post-processing when capturing in RAW mode. Lighting can have a critical influence on the way objects are captured in digital images. Images taken in the lab can be done with controlled lighting such as artificial lighting sources. However, analysis of images collected in the field must compensate for variable lighting conditions. Overcast skies provide a good lighting source for collecting digital images in the field. Bright sun should be avoided where possible as heavy shadows can be cast. A portable diffuser (light umbrella) can be used to reduce the effects of direct sun. A colour reference should be included in all images to allow careful colour balancing in post-processing (in this study, a ColorChecker card was used). A critical issue for recording bright colours is channel clipping due to image overexposure [34]. When field measurements are taken under difficult lighting conditions (e.g. direct sun with strong shadows), the dynamic range of the available light may exceed the range of the camera sensor, and RGB values may not be recorded accurately. Raw RGB values will be limited to their maximum value (typically 255) meaning that transformation into L*, a* and b* values will be inaccurate. In this situation, the exposure of the photograph should be adjusted to ensure that the colour of interest is not clipped (many cameras have a review function that highlights clipped areas of the image).

## Supporting Information

File S1 **In-situ photographs of the nine yellow flowering species and models of the two invasive *Hieracium* species used in the experiment.**
(TIF)

File S2 **Photograph of a 1m^2^ quadrat showing the yellow flowering *Craspedia* *aurantia* *- C. jamesii* complex.**
(TIF)

File S3 **Leaves of the five species collected to compare camera reliability.**
(TIF)

File S4 R code to extract CIE 1976 (L*a*b*) values from TIF file.
(DOC)

File S5 L*, a* and b* histograms of flowers used in the study. The recorded distribution is shown with black circles, and the modelled distribution (Gaussian using mean and sd) is shown as a grey line.
(TIF)

File S6 L*, a* and b* histograms of leaves used in the study. The recorded distribution is shown with black circles, and the modelled distribution (Gaussian using mean and sd) is shown as a grey line.
(TIF)
