# Supplementary figures and images for: Quantifying Plant Colour and Colour Difference as Perceived by Humans Using Digital Images
Source: PLoS One. 2013 Aug 20;8(8):e72296. doi: 10.1371/journal.pone.0072296 (PMC3748102; doi:10.1371/journal.pone.0072296)

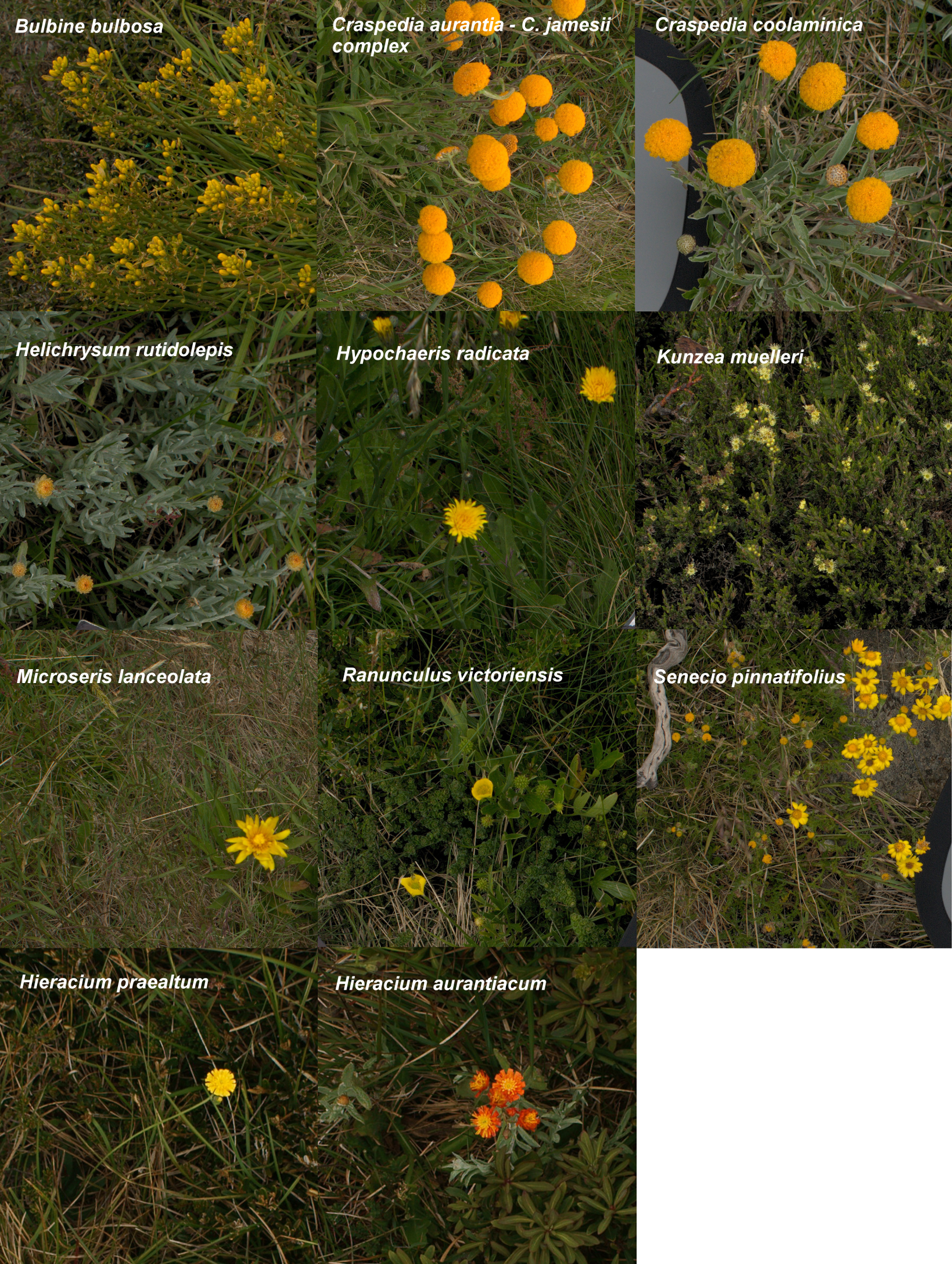

Supplement: File S1 — In-situ photographs of the nine yellow flowering species and models of the two invasive Hieracium species used in the experiment. (TIF) [file pone.0072296.s001.tif]

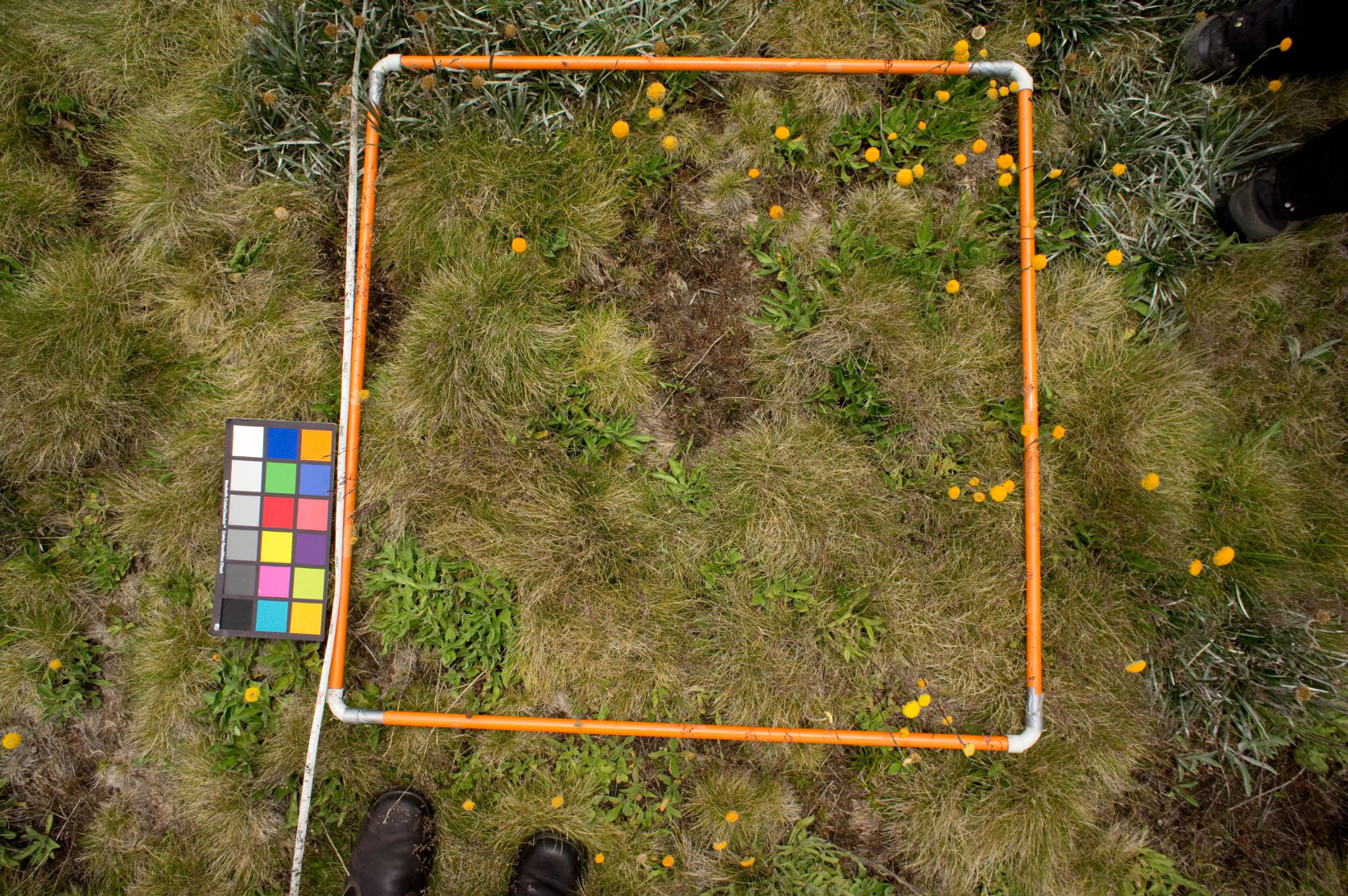

Supplement: File S2 — Photograph of a 1m2 quadrat showing the yellow flowering Craspedia aurantia - C. jamesii complex. (TIF) [file pone.0072296.s002.tif]

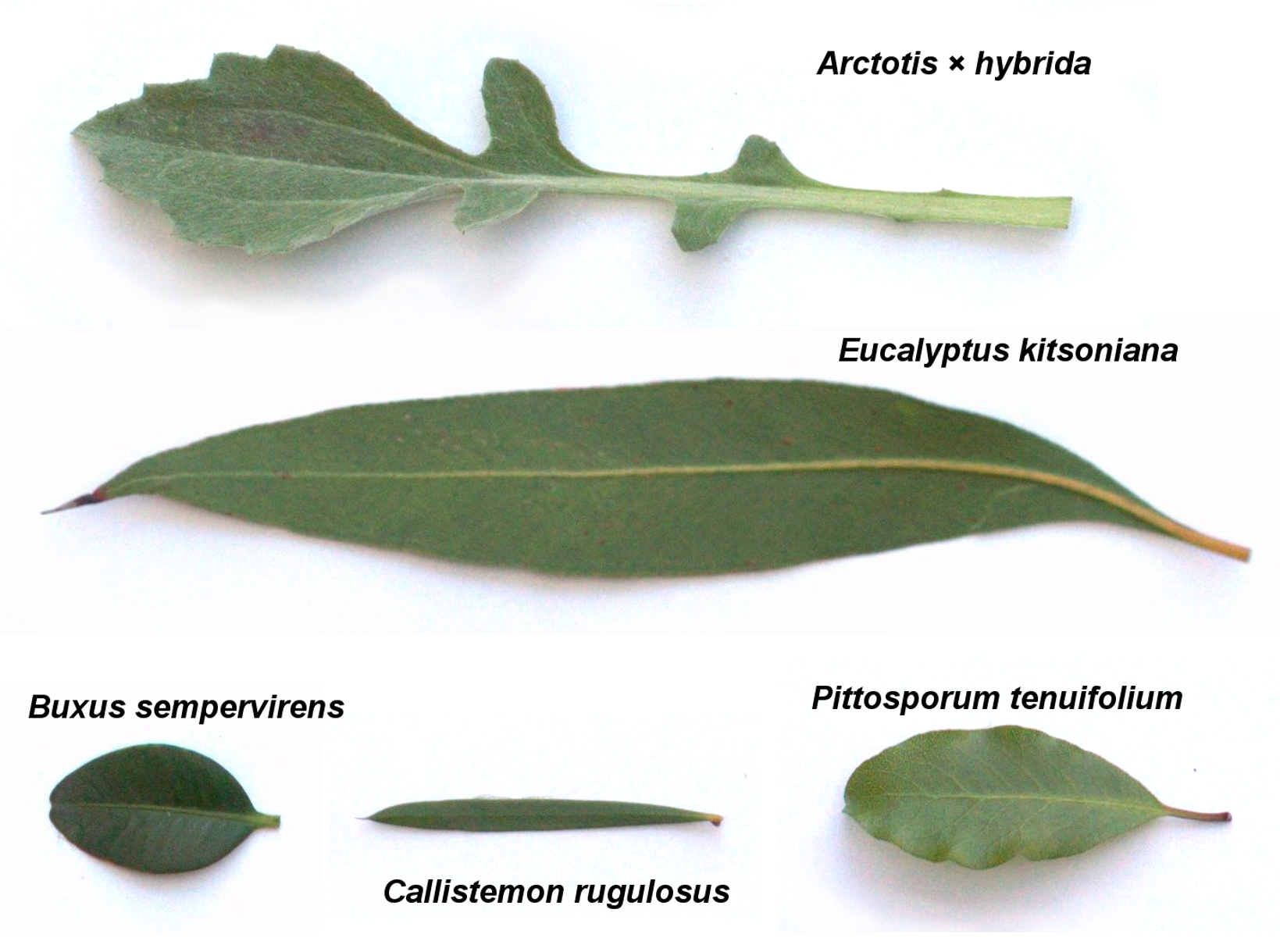

Supplement: File S3 — Leaves of the five species collected to compare camera reliability. (TIF) [file pone.0072296.s003.tif]

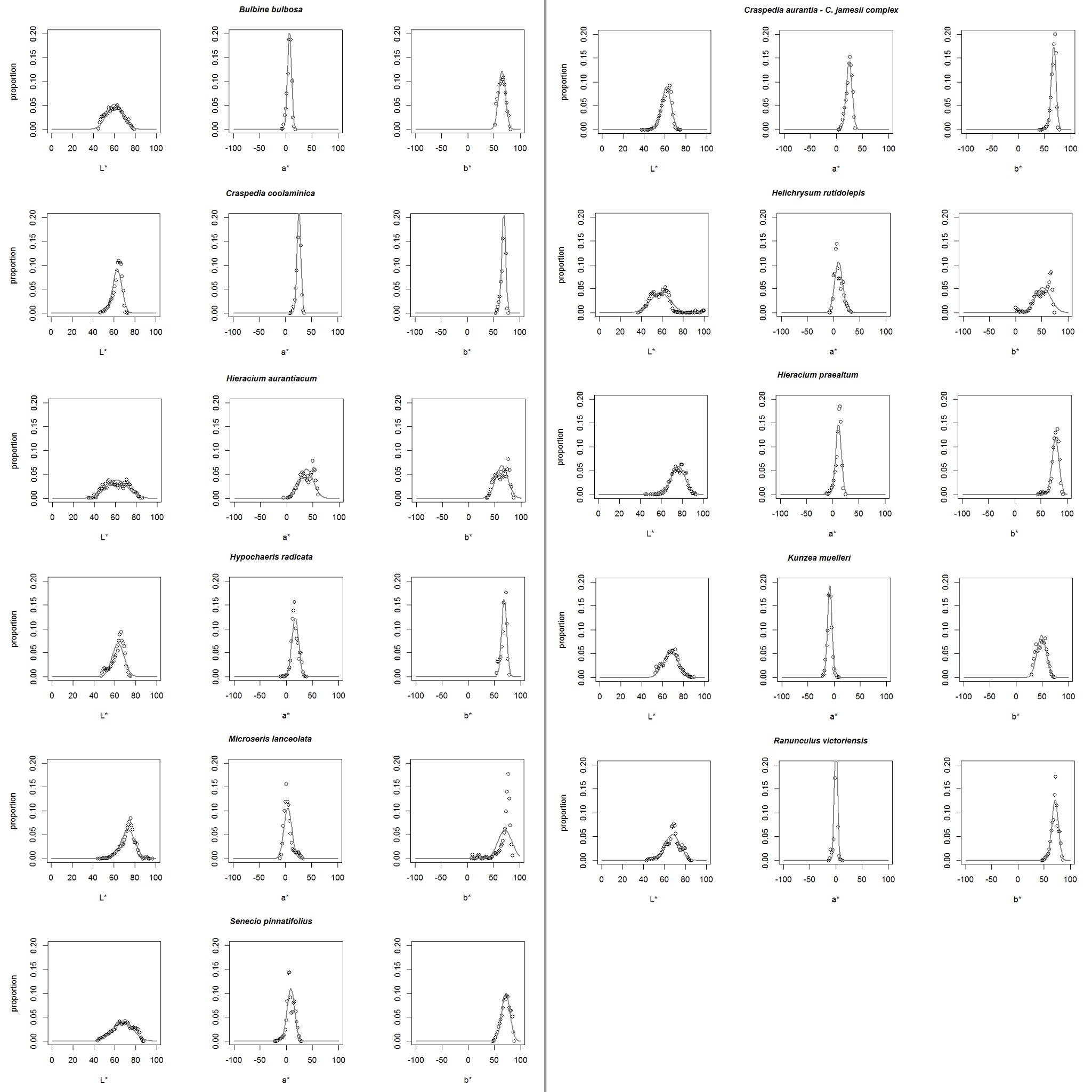

Supplement: File S5 — L*, a* and b* histograms of flowers used in the study. The recorded distribution is shown with black circles, and the modelled distribution (Gaussian using mean and sd) is shown as a grey line. (TIF) [file pone.0072296.s005.tif]

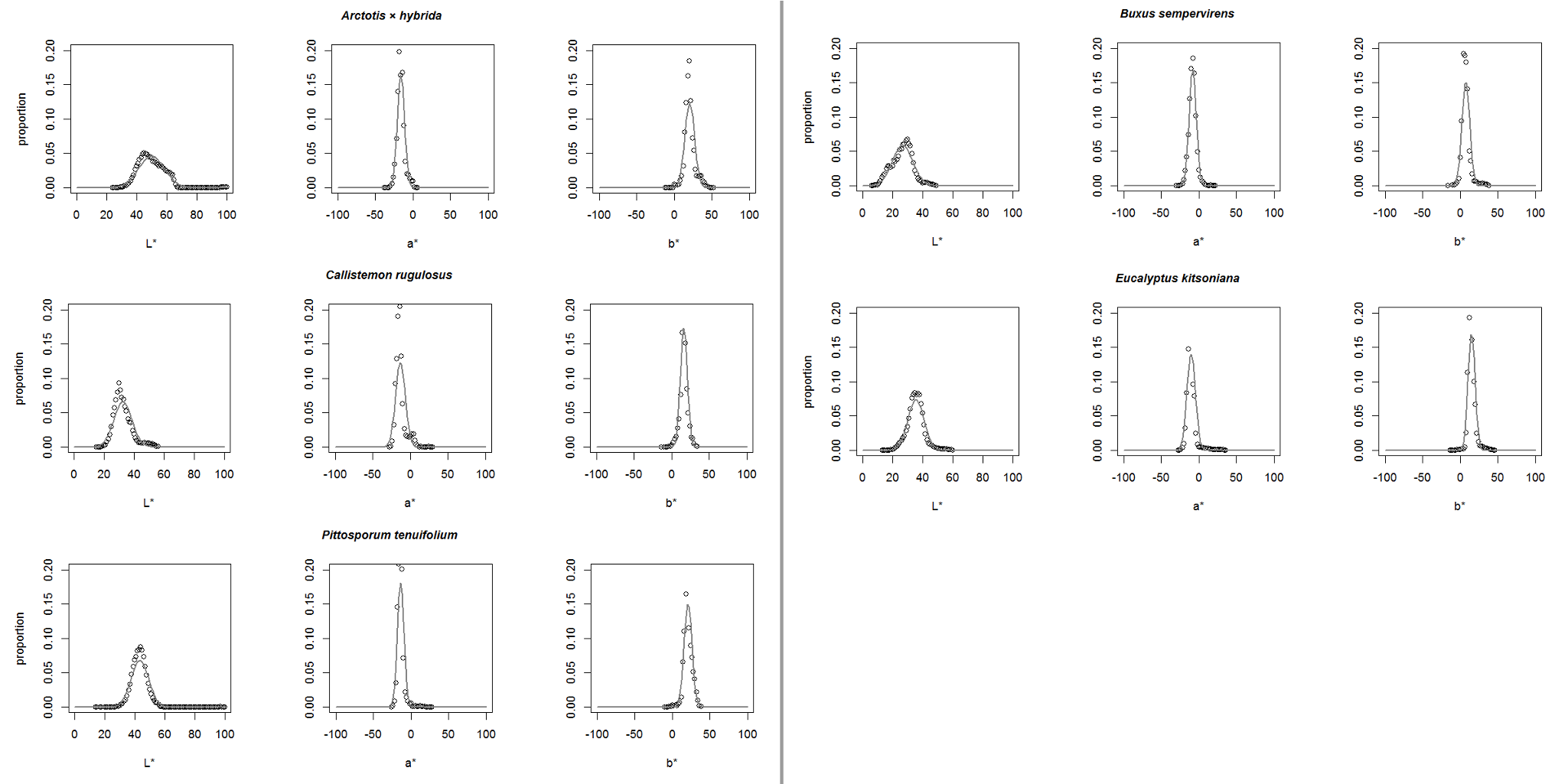

Supplement: File S6 — L*, a* and b* histograms of leaves used in the study. The recorded distribution is shown with black circles, and the modelled distribution (Gaussian using mean and sd) is shown as a grey line. (TIF) [file pone.0072296.s006.tif]
