# Supplementary material for: Quantifying Plant Colour and Colour Difference as Perceived by Humans Using Digital Images
Source: PLoS One. 2013 Aug 20;8(8):e72296. doi: 10.1371/journal.pone.0072296 (PMC3748102; doi:10.1371/journal.pone.0072296)
Supplement: File S4 — R code to extract CIE 1976 (L*a*b*) values from TIF file. (DOC) [file pone.0072296.s004.doc]

**Quantifying plant colour and colour difference as perceived by humans using digital images**

**Supporting information file S4**

R code to extract CIE 1976 (*L*a*b**) values from TIF file

# change this to your working directory

setwd("C:/Data")

## load image file

library(rtiff)

# change this to your image file

pic.tif <- readTiff("image.tiff")

## convert tiff to CIE 1976 (L*a*b*)

pic.rgb <- data.frame( red=c(pic.tif@red),

green=c(pic.tif@green),

blue=c(pic.tif@blue) )

pic.Lab <- convertColor( pic.rgb,from="sRGB", to="Lab", clip=NA )

# this assumes an image where the background is white,

# and there are no fully saturated blues in the object

mask <- (pic.tif@blue<1)

## calculate descriptive statistics

# means

L.mean <- mean(pic.Lab[mask,iL])

a.mean <- mean(pic.Lab[mask,ia])

b.mean <- mean(pic.Lab[mask,ib])

# sds

L.sd <- sd(pic.Lab[mask,iL])

a.sd <- sd(pic.Lab[mask,ia])

b.sd <- sd(pic.Lab[mask,ib])
